# Vitamin D Deficiency Mediates the Link Between Dietary Patterns, Inflammatory Biomarkers, and Iron Status Indicators (Ferritin and Hemoglobin) in Metabolic Syndrome

**DOI:** 10.3390/nu18020224

**Published:** 2026-01-10

**Authors:** Salma I. Cortes-Álvarez, Iván Delgado-Enciso, Gustavo A. Hernández-Fuentes, José Guzmán-Esquivel, Janet Diaz-Martinez, Alejandrina Rodríguez-Hernández, Margarita L. Martinez-Fierro, Iram P. Rodríguez-Sánchez, Valery Melnikov, Yunue Flores-Ruelas, Idalia Garza-Veloz, Miriam De la Cruz-Ruiz, Ángel A. Ramos-Organillo, Carmen A. Sánchez-Ramírez

**Affiliations:** 1 Department of Molecular Medicine, School of Medicine, University of Colima, Colima 28040, Mexico; scortes4@ucol.mx (S.I.C.-Á.); gahfuentes@gmail.com (G.A.H.-F.); arodrig@ucol.mx (A.R.-H.); valery.melnikoff@gmail.com (V.M.); 2 Colima State Institute of Cancerology, IMSS-Bienestar, Colima 28085, Mexico; 3 Research Center in Minority Institutions, Robert Stempel College of Public Health, Florida International University, Miami, FL 33199, USA; jdimarti@fiu.edu; 4 Faculty of Chemical Sciences, University of Colima, Coquimatlan 28400, Mexico; aaramos@ucol.mx; 5 Clinical Epidemiology Research Unit, Mexican Institute of Social Security, Villa de Alvarez, Colima 28984, Mexico; jose.esquivel@imss.gob.mx; 6 Molecular Medicine Laboratory, Academic Unit of Human Medicine and Health Sciences, Autonomous University of Zacatecas, Zacatecas 98160, Mexico; margaritamf@uaz.edu.mx (M.L.M.-F.); idaliagv@uaz.edu.mx (I.G.-V.); 7 Molecular and Structural Physiology Laboratory, School of Biological Sciences, Universidad Autónoma de Nuevo León, San Nicolás de los Garza 66455, Mexico; iramrodriguez@gmail.com; 8 Institute of Human Nutrition, University Center for Health Sciences, University of Guadalajara, Salvador Quevedo y Zubieta No. 750, Hospital Civil de Guadalajara, Annex Building, 3rd Floor, Independencia Neighborhood, Guadalajara 44340, Mexico; yun.ruelas@gmail.com; 9 Oficina de Investigación en Salud, Servicios de Salud del Instituto Mexicano del Seguro Social Para el Bienestar (IMSS-BIENESTAR), Colima 28085, Mexico; miriam.delacruz@imssbienestar.gob.mx

**Keywords:** vitamin D deficiency, metabolic syndrome (MetS), C-reactive protein (CRP), inflammation, dietary vitamin D intake, ferritin, neutrophil-to-lymphocyte ratio (NLR), obesity, nutritional epidemiology, hyperferritinemia, acute-phase reactants, hemoglobin, mediation analysis

## Abstract

**Background/Objectives**: Chronic low-grade inflammation and nutritional deficiencies, particularly vitamin D deficiency, have emerged as important contributors to Metabolic syndrome (MetS) pathogenesis but remain underexplored. This study aimed to comprehensively evaluate the associations between dietary intake, vitamin D status, and inflammatory biomarkers (high-sensitivity C-reactive protein -CRP- and ferritin) in patients with MetS. **Methods**: A cross-sectional observational study was conducted on 141 adult MetS patients at a Mexican hospital. Clinical, anthropometric, dietary (using a validated food frequency questionnaire), and biochemical data including serum 25-hydroxyvitamin D, CRP, ferritin, and neutrophil-to-lymphocyte ratio (NLR) were collected. Vitamin D deficiency was defined as serum 25(OH)D < 20 ng/mL, and high inflammation as CRP ≥ 3 mg/L. Logistic regression models adjusted for confounders were used to analyze associations. Mediation analysis assessed whether vitamin D deficiency mediated the link between dietary intake and high CRP or ferritin. **Results**: Patients with elevated CRP had significantly lower serum vitamin D levels (14.0 ± 5.1 vs. 22.1 ± 7.0 ng/mL; *p* < 0.001). Multivariable analysis showed vitamin D deficiency (adjusted OR 7.1; 95% CI 2.5–19.4; *p* < 0.001) and hyperferritinemia (ferritin ≥ 200 μg/L; aOR 8.0, 95% CI 3.5–18.2, *p* < 0.001) as predictors of high CRP. Conversely, hyperferritinemia was predicted by vitamin D deficiency (aOR 24.69; 95% CI 3.76–162.16; *p* = 0.001), elevated CRP (aOR 5.06; *p* = 0.014), Hb (aOR 63.23; *p* < 0.001), and inversely by grade 2 obesity (aOR 0.11; 95% CI 0.02–0.60; *p* = 0.03), confirming bidirectional CRP-ferritin associations and hyperferritinemia as an inflammation marker rather than iron overload indicator. Although Hb > 14.3 g/dL associated with hyperferritinemia, it did not independently predict CRP in multivariate analyses. Frequent consumption of vitamin D-rich foods (milk, fish, Manchego and Oaxaca cheese) was associated with lower inflammation. Mediation analysis confirmed that vitamin D deficiency mediated dietary intake-CRP and dietary intake-ferritin links (Sobel test *p* < 0.05). **Conclusions**: Vitamin D deficiency is a key mediator linking inadequate dietary vitamin D intake to systemic inflammation in MetS. Nutritional strategies emphasizing vitamin D repletion and consumption of vitamin D fortified foods may effectively reduce chronic inflammation and improve metabolic outcomes.

## 1. Introduction

Metabolic syndrome (MetS) is a cluster of interrelated metabolic abnormalities, including central obesity, dyslipidemia, hypertension, and insulin resistance, that significantly increases the risk of cardiovascular disease, type 2 diabetes, and other chronic conditions [1]. The global prevalence of MetS continues to rise, largely driven by sedentary lifestyles, poor dietary patterns, and increasing rates of obesity, reflecting major public health challenges worldwide [2,3]. Although traditional clinical markers such as waist circumference, triglyceride levels, and HDL cholesterol have been extensively studied and are central to MetS diagnosis, emerging evidence underscores the crucial role of chronic low-grade inflammation and nutritional deficiencies in its pathogenesis and progression [4,5,6].

In recent years, advances in biomarker research have identified C-reactive protein (CRP) and ferritin as reliable indicators of subclinical inflammation in patients with MetS [7,8,9,10,11]. Elevated levels of these biomarkers have been associated with increased cardiovascular risk and metabolic dysfunction, offering valuable insight into the underlying inflammatory state of affected individuals [7,8]. Parallel to this, vitamin D deficiency has gained increasing attention as a modifiable risk factor for MetS. Numerous studies have demonstrated strong associations between low vitamin D levels and key components of MetS, including central obesity, insulin resistance, and dyslipidemia [12,13,14,15].

Despite these scientific advances, important knowledge gaps persist regarding the complex interplay between nutritional patterns, dietary intake, and inflammatory biomarkers in MetS. Most existing research has focused on isolated nutrients or single biomarkers, providing only limited understanding of how dietary habits, anthropometric measures, and metabolic parameters interact to influence systemic inflammation. Addressing this limitation requires comprehensive evaluation of nutritional and inflammatory profiles in affected populations [16,17,18,19].

The present study aims to contribute to this understanding by examining the associations between emerging inflammatory biomarkers—specifically high-sensitivity C-reactive protein (hs-CRP) and ferritin—and nutritional factors in a cohort of patients with MetS. We hypothesize that specific dietary patterns and nutritional deficiencies, particularly vitamin D deficiency, are independently associated with elevated inflammatory markers. Furthermore, given that chronic low-grade inflammation is a key driver of metabolic dysfunction, elucidating these relationships may provide clinically relevant information to guide targeted nutritional interventions. By integrating clinical, nutritional, and biochemical data, this study seeks to address critical knowledge gaps and enhance the current understanding of the metabolic and inflammatory mechanisms underlying MetS. Ultimately, these findings may support the development of evidence-based nutritional strategies aimed at improving inflammatory profiles and clinical outcomes in patients with MetS.

## 2. Materials and Methods

### 2.1. Study Design and Setting

This cross-sectional observational correlational study was conducted at the General Zone Hospital No. 1 of the “Instituto Mexicano del Seguro Social” (IMSS) in Villa de Álvarez, Colima, from January to December 2023. The study received approval from the Institutional Ethics Committee (approval number R-2021-601-019; 23 August 2021) and adhered to national and international ethical standards, including the Declaration of Helsinki and the STROBE guidelines for transparent reporting of observational studies [20].

### 2.2. Population and Sample

Participant recruitment was carried out in the Endocrinology outpatient clinic using consecutive sampling. Patients with a confirmed diagnosis of metabolic syndrome, defined according to the harmonized NCEP ATP-III criteria requiring the presence of at least three of the five established metabolic components, were screened for eligibility [21,22].

Metabolic syndrome was diagnosed when at least three of the following five components were present: (1) Abdominal obesity: waist circumference ≥ 102 cm in men or ≥88 cm in women. (2) Hypertriglyceridemia: serum triglycerides ≥ 150 mg/dL or current treatment for elevated triglycerides. (3) Low HDL cholesterol: <40 mg/dL in men or <50 mg/dL in women, or current treatment for reduced HDL cholesterol. (4) Elevated blood pressure: systolic blood pressure ≥ 130 mmHg and/or diastolic blood pressure ≥ 85 mmHg, or current use of antihypertensive medication and (5) Impaired fasting glucose: fasting plasma glucose ≥ 100 mg/dL or previously diagnosed type 2 diabetes mellitus [23,24].

Inclusion criteria were adults ≥ 18 years old and the ability to provide written informed consent. Exclusion criteria included: active infectious diseases, chronic inflammatory conditions other than MetS, active cancer, advanced renal or hepatic failure, recent use of medications affecting vitamin D or iron metabolism, current supplementation with these micronutrients, and incomplete participation in the study procedures. Of the 220 individuals screened, 141 met all inclusion criteria and were included in the final analysis.

### 2.3. Clinical and Anthropometric Data Collection

Clinical data was obtained through structured interviews and review of recent medical records. Body weight was measured using a SECA^®^ (Hamburg, Germany) digital clinical scale (capacity 250 kg; resolution 100 g) calibrated at the start of each measurement session. Height was measured with a fixed SECA^®^ stadiometer with 1 mm precision following a standardized protocol, with participants standing upright, barefoot, and wearing light clothing. Waist circumference was measured using a non-stretchable SECA^®^ measuring tape (length approx. 150 cm; accuracy 1 mm), positioned at the midpoint between the lowest rib and the iliac crest, at the end of a normal expiration. Blood pressure was measured with a clinically validated Omron^®^ (Kyoto, Japan) HEM-907 digital sphygmomanometer. Measurements were taken after at least 5 min of rest, using an appropriately sized cuff, and averaged from two readings taken 1–2 min apart. All anthropometric measurements were performed by trained personnel following the standards of the International Society for the Advancement of Kinanthropometry (ISAK) [25]. Sociodemographic information, lifestyle habits, medical history, and medication use were also recorded.

### 2.4. Dietary Assessment: SNUT Questionnaire

Habitual dietary intake was assessed using the semiquantitative SNUT Food Frequency Questionnaire (“Sistema de Evaluación del Consumo Alimentario”), a tool validated in the Mexican population by the National Institute of Public Health (“Instituto Nacional de Salud Pública,” INSP) for estimating long-term consumption of foods and nutrients [26]. The questionnaire includes 112 items covering major food groups, including relevant sources of vitamin D and iron [26].

Administration was conducted in person by trained staff following standardized procedures to minimize recall bias. Visual aids (cards with photographs of standard portion sizes) were used to improve portion estimation accuracy [27,28,29].

Dietary data were processed using the SNUT^®^ version 3.0 software, which calculates daily and monthly intake of macronutrients (carbohydrates, proteins, fats) and micronutrients (vitamins and minerals). The system incorporates internal algorithms for detecting inconsistencies and standardizing intakes, ensuring data quality and reproducibility [30].

### 2.5. Assessment of Sun Exposure and Photoprotection

Sun exposure and photoprotection habits were evaluated with the Sun Exposure and Protection Index (SEPI) questionnaire, which quantifies frequency, duration, and patterns of sunlight exposure, as well as sunscreen use, clothing, hats, and other protective measures [31,32]. A higher score generally indicates greater exposure to sunlight. The SEPI has demonstrated validity in Mexican adults and allows categorization of participants into different levels of sun exposure and protection [33].

For analysis, total SEPI scores were dichotomized using the median (50th percentile) as a cutoff to classify participants into low vs. high exposure/protection groups. This evaluation is essential for interpreting vitamin D status since cutaneous synthesis is the primary endogenous source of this vitamin [34].

### 2.6. Biological Sample Collection and Laboratory Analyses

Fasting venous blood samples were collected from all participants after an overnight fast of at least 8 h. Samples for complete blood count analysis were obtained in EDTA-containing tubes and processed immediately to determine hematological parameters required for calculating the neutrophil-to-lymphocyte ratio (NLR) as an inflammatory marker [35]. Samples intended for biochemical and immunoassay analyses were collected in serum separator tubes or plasma tubes (lithium heparin or EDTA), according to the requirements of each assay. After collection, samples were allowed to clot when applicable and were centrifuged at 3000× *g* for 10 min to obtain serum or plasma.

To preserve the stability of 25-hydroxyvitamin D [25(OH)D], preanalytical handling strictly followed the manufacturer’s recommendations. Serum or plasma samples were protected from direct light to prevent analyte degradation. When analyses were performed on the same day, samples were stored at 2–8 °C until processing. For delayed analyses, samples were stored frozen at −20 °C or −70 °C, depending on storage duration, and analyzed within the timeframes specified by the manufacturer to ensure analyte stability. Serum 25-hydroxyvitamin D concentrations were measured using a fully automated electrochemiluminescence immunoassay (ECLIA) with the Elecsys^®^ Vitamin D total III assay (Roche Diagnostics) on a cobas e 801 analyzers.

Serum ferritin concentrations were determined using the Elecsys^®^ Ferritin assay (Roche Diagnostics, Indianapolis, IN, USA; ACN FERR 10034) on cobas e immunoassay analyzers (cobas e411 or cobas e 601), depending on instrument availability. Both assays were performed strictly in accordance with the manufacturer’s instructions, including calibration procedures. The analytical performance of these assays, as verified through routine internal and external quality control procedures, supports the reporting of most biochemical parameters to two decimal places.

C-reactive protein (CRP) levels were quantified in serum using an automated immunoturbidimetric method on a Cobas C503 analyzer (Roche Diagnostics), following standardized laboratory operating procedures [36].

All biochemical and immunoassay analyses were conducted in the clinical laboratory of the General Zone Hospital No. 1 of the Instituto Mexicano del Seguro Social (IMSS) [37]. The laboratory operates under institutional standardized procedures for internal quality assurance. Internal quality control was routinely performed using manufacturer-provided control materials (PreciControl^®^, Roche Diagnostics) at multiple concentration levels prior to sample analysis. In addition, the laboratory participates in national external quality assessment programs (Qualitat^®^), which allow continuous monitoring of analytical performance through interlaboratory comparisons [38,39,40].

Measurement uncertainty (MU) was not estimated separately for this study, as it was designed as a clinical-epidemiological investigation rather than an analytical method validation. Nevertheless, analytical variability was routinely monitored and maintained within acceptable performance limits defined by the manufacturer and verified through internal and external quality control procedures [41,42].

Vitamin D deficiency was defined as serum 25(OH)D levels < 20 ng/mL. Serum ferritin concentrations > 200 µg/L, CRP levels ≥ 3 mg/L, or an NLR > 3.5 were considered indicative of systemic inflammation, according to established clinical criteria [43,44,45,46]. All tests reported in this study fall within the laboratory’s routine clinical testing scope and are performed under established institutional protocols, ensuring the accuracy, precision, and reproducibility of the reported results [47].

### 2.7. Statistical Analysis

Normality was assessed using the Kolmogorov–Smirnov test. Descriptive continuous variables were presented as means ± standard deviations, and categorical variables as frequencies and proportions. The 50th percentile (median) of the SEPI score was used to dichotomize the population. Group comparisons were performed using Student’s *t*-test for continuous variables and Fisher’s exact test for categorical variables [48].

Crude odds ratios (ORs) with 95% confidence intervals were initially calculated using bivariate logistic regression to explore unadjusted associations with elevated CRP levels. Subsequently, a multivariate binary logistic regression model with backward stepwise selection was applied, using significance thresholds of 0.05 for entry and 0.10 for retention; only the most parsimonious final model is presented. Adjustments were made for age, sex, BMI, and other confounding variables to identify independent associations and potential mediating effects.

Receiver Operating Characteristic (ROC) curve analysis was conducted to assess the predictive performance of monthly total vitamin D intake for serum vitamin D deficiency. The area under the curve (AUC), 95% confidence intervals, *p*-values, and the optimal cut-off point (defined as the threshold providing the best balance between sensitivity and specificity) were reported. Additionally, this analysis was also used to determine the hemoglobin cutoff point that predicts a CRP > 3 mg/L.

To investigate whether serum vitamin D deficiency mediates the relationship between low dietary vitamin D intake and elevated C-reactive protein (CRP) levels, a mediation analysis was performed using logistic regression. Two models were constructed: Model A: estimated the effect of dietary vitamin D intake on serum vitamin D deficiency, adjusting for demographic and clinical covariates. Model B: estimated the effect of vitamin D deficiency on elevated CRP levels, adjusting for vitamin D intake and confounders.

The indirect (mediated) effect was calculated using the Sobel method, based on coefficients and standard errors from both models. Statistical significance was determined using Sobel test *p*-values.

All analyses were conducted using SPSS version 25 (IBM Corp., Armonk, NY, USA). Sample size was calculated with OpenEpi version 1 (https://www.openepi.com/SampleSize/SSCC.htm, accessed on 15 April 2025) [49], statistical power was calculated using ClinCalc version 1 (https://clincalc.com/stats/Power.aspx, accessed on 30 November 2025; https://quantpsy.org/sobel/sobel.htm, accessed on 30 November 2025) [50], and the Sobel test was performed using the online tool by Kristopher J. Preacher (https://quantpsy.org/sobel/sobel.htm, accessed on 15 September 2025) [51].

The significance level of *p* < 0.05 was considered statistically significant. All procedures followed the STROBE recommendations to ensure methodological rigor and transparency in reporting [52].

## 3. Results

### 3.1. Sample Characteristics and Inflammation Status

The study cohort included 141 metabolic syndrome patients with mean age 49.2 years and predominance of women (68.1%). Stratification by elevated C-reactive protein (CRP) revealed patients with higher systemic inflammation had significantly higher BMI (35.4 vs. 32.1, *p* = 0.013) and hemoglobin -Hb- (14.5 vs. 13.7, *p* < 0.00), but markedly lower mean serum vitamin D levels (14.0 vs. 22.1 ng/mL, *p* < 0.001) compared to those with normal CRP (Table 1). Dietary patterns also differed, showing lower consumption of vitamin D-rich and fortified foods such as milk, Oaxaca cheese, and yogurt among patients with high CRP (Table 2). These findings support a nutritional phenotype potentially contributing to chronic inflammation within metabolic syndrome beyond classical metabolic risk factors. It is important to note that only 2.8% of the subjects had Hb values consistent with anemia (<13.5 g/dL in men; <12.0 g/dL in women) [53], rendering this a clinically irrelevant factor in the analyzed sample.

### 3.2. Risk Factors Associated with Elevated C-Reactive Protein

Among patients with elevated C-reactive protein levels (CRP > 3 mg/L), 89.1% exhibited serum vitamin D deficiency (<20 ng/mL), whereas only 33.8% of patients with CRP levels below 3 mg/L presented this deficiency. In bivariate analyses, vitamin D deficiency was strongly associated with elevated CRP levels, yielding an odds ratio (OR) of 15.97 (95% confidence interval [CI] 6.39–39.92; *p* < 0.001). Elevated ferritin levels were also strongly associated with increased CRP levels. (OR 10.65, 95% CI 4.84–23.46; *p* < 0.001). Obesity grade 2 or higher showed a significant association with elevated CRP levels (OR 2.44, 95% CI 1.16–5.15; *p* = 0.019). Elevated total cholesterol (>200 mg/dL) also was associated with high CRP (OR 8.06, 95% CI 3.11–20.89; *p* < 0.001), as did a neutrophil-to-lymphocyte ratio (NRL) greater than 3.5 with high CRP (OR 2.76; 95% CI 1.26–6.05; *p* = 0.011). No significant associations were observed for sex, age, or physical activity in this unadjusted model. Since 97% of patients had normal Hb levels and dichotomizing the sample solely by the presence/absence of anemia would be uninformative, the association analysis with Hb levels required prior receiver operating characteristic (ROC) curve analysis to predict CRP > 3 mg/L. In women and men, the AUC for predicting CRP > 3 mg/L was 0.879 (95% CI 0.809–0.950; *p* < 0.001) and 0.823 (95% CI 0.705–0.941; *p* < 0.001), respectively, with an AUC difference between sexes of 0.056 (*p* = 0.427). Without considering sex, the AUC was 0.826 (95% CI 0.757–0.895, *p* < 0.001) with a cutoff point of 14.3 g/dL (sensitivity = 70.31%; specificity = 81.82%; positive predictive value = 76.28%; negative predictive value = 76.82%; accuracy = 76.6%). In bivariate analyses, Hb > 14.3 g/dL was strongly associated with elevated CRP levels, yielding an odds ratio (OR) of 10.65 (95% CI 4.84–23.46; *p* < 0.001). After adjusting for confounding variables, only vitamin D deficiency and serum ferritin levels ≥ 200 mg/L remained robust independent predictors of elevated CRP (adjusted OR [aOR] 7.11 and 8.00, respectively; both *p* < 0.001; see Table 3). It is noteworthy that Hb > 14.3 g/dL was not associated with high CRP in the multivariable analysis, whereas elevated ferritin levels remained significantly associated (Table 3).

Serum vitamin D deficiency prevalence differed significantly between groups defined by C-reactive protein (CRP) thresholds. Specifically, 89.1% of patients with CRP > 3 mg/L exhibited vitamin D deficiency (<20 ng/mL), compared to 33.8% among those with CRP < 3 mg/L. A post hoc power analysis for this comparison of two proportions indicated the study had approximately 99% power to detect this difference, confirming robustness and adequate sample size for the observed significant association.

### 3.3. Predictors of Hyperferritinemia

Among patients with serum ferritin levels ≥ 200 mg/L, 90.0% exhibited vitamin D deficiency (<20 ng/mL), compared to only 35.8% deficiency prevalence among those with ferritin levels below 200 mg/L. In this sample, vitamin D deficiency was strongly associated with hyperferritinemia (OR 16.14, 95% CI 6.19–42.06; *p* < 0.001), as were elevated CRP (OR 16.25, 95% CI 6.19–42.06; *p* < 0.001) and Hb (OR 51.70, 95% CI 18.69–143.01; *p* < 0.001). Elevated cholesterol (>200 mg/dL) also showed a significant association with hyperferritinemia (OR 16.79; 95% CI 4.86–58.04; *p* < 0.001), as did elevated NRL with hyperferritinemia (OR 2.37; 95% CI 1.09–5.13; *p* = 0.028). Body mass index grade 2 or higher did not show a positive bivariate association. In the adjusted model, vitamin D deficiency remained significantly associated with hyperferritinemia (aOR 24.69; 95% CI 3.76–162.16; *p* = 0.001), as did elevated CRP (aOR 5.06; *p* = 0.014) and Hb (aOR 63.23; *p* < 0.001). Interestingly, obesity grade 2 was inversely associated with hyperferritinemia (aOR 0.11; 95% CI 0.02–0.60; *p* = 0.03), suggesting complex metabolic-inflammation interplay (Table 4).

### 3.4. Nutritional and Lifestyle Factors Associated with Vitamin D Deficiency

Obesity grade 2 or higher significantly increased odds of vitamin D deficiency (OR 4.12, 95% CI 1.73–9.81; *p* = 0.001). Frequent consumption of fortified dairy foods (milk, manchego cheese) and fish were protective against vitamin D deficiency. Fish intake was protective, with a markedly reduced risk (OR 0.07; *p* < 0.001). In the final multivariable model (Table 5), high SEPI score (protective; aOR 0.38; *p* = 0.033), milk (protective; aOR 0.37; *p* = 0.040), manchego cheese (protective; aOR 0.20; *p* = 0.010), butter (risk factor; aOR 4.36; *p* = 0.027), and fish (protective; aOR 0.15; *p* = 0.001) were independently associated with vitamin D deficiency. Obesity grade 2 did not retain significance after adjustment (Table 5).

The monthly total vitamin D intake was a significant predictor of serum vitamin D deficiency, with an area under the curve (AUC) of 0.180 (95% CI 0.107 to 0.253, *p* < 0.001). The optimal cut-off point was determined to be 3281 IU, which provided the best balance between sensitivity (69.88%) and specificity (70.69%). Among patients with a monthly vitamin D intake below 3281 IU, 77.3% exhibited serum vitamin D deficiency (<20 ng/mL), whereas only 37.9% of those with intake above this threshold presented deficiency. Low monthly vitamin D intake was strongly associated with deficiency (crude odds ratio 5.59, 95% confidence interval 2.68–11.66, *p* = 0.001).

### 3.5. Mediation Analysis

Mediation analysis revealed that low vitamin D intake was strongly associated with serum vitamin D deficiency (coefficient B = 2.036, OR = 7.662, 95% CI 2. 2.669–21.995, *p* < 0.001). In turn, vitamin D deficiency was significantly associated with elevated CRP levels, adjusted for dietary intake (coefficient B = 2.462, OR = 11.731, 95% CI 2.603–52.875, *p=* 0.001). The direct effect of dietary intake on CRP adjusted for vitamin D deficiency was not statistically significant (B = −0.616, OR = 0.54, *p* = 0.203), indicating that the primary association occurs via the mediator. The indirect effect, calculated by the Sobel test, was significant (a × b = 2.446, *p* = 0.014), confirming that serum vitamin D deficiency mediates the relationship between dietary intake and systemic inflammation as estimated by CRP (Table 6).

Given the robust bivariate (OR 16.14, *p* < 0.001) and multivariate (aOR 24.69, *p* = 0.001) association between vitamin D deficiency and hyperferritinemia (Table 4), we evaluated serum vitamin D deficiency as a potential mediator between deficient dietary vitamin D intake and elevated ferritin levels. Low vitamin D intake strongly predicted vitamin D deficiency (B = 2.036, OR = 7.662, 95% CI 2.669–21.995, *p* < 0.001). Vitamin D deficiency significantly associated with hyperferritinemia after dietary adjustment (B = 3.013, OR = 20.357, 95% CI 3.181–130.255, *p* = 0.001), while the direct dietary intake to ferritin effect was non-significant (B = −0.517, OR = 0.597, *p* = 0.272), confirming complete mediation. The indirect effect was significant (Sobel test: a × b=2.435, *p* = 0.014) (Table 7).

The mediation analysis that assessed serum ferritin as a mediator between elevated hemoglobin and C-reactive protein (CRP) levels in individuals with metabolic syndrome confirmed a significant indirect effect (Sobel test z = 3.373, *p* < 0.001; Table 8). Elevated hemoglobin strongly predicted hyperferritinemia (Model A: OR 63.24, 95% CI 13.01–307.50, *p* < 0.001), which in turn independently predicted elevated CRP (Model B: OR 8.00, 95% CI 3.21–19.92, *p* < 0.001). The direct effect of hemoglobin on CRP, adjusted only for ferritin, was not significant (OR 2.54, 95% CI 0.85–7.65, *p* = 0.097), confirming complete mediation via the ferritin pathway and demonstrating that inflammation is driven by ferritin, not directly by hemoglobin.

These three mediation analyses collectively demonstrate interconnected inflammatory mechanisms in metabolic syndrome. Vitamin D deficiency serves as a central mediator linking dietary intake to both CRP elevation (Table 6) and hyperferritinemia (Table 7), while ferritin independently mediates the hemoglobin-CRP relationship (Table 8). Despite shared pathways, vitamin D deficiency and ferritin emerge as complementary, independent predictors of systemic inflammation in the final multivariate model (Table 3), highlighting their distinct yet interrelated roles in MetS pathophysiology (all Sobel *p* < 0.05).

## 4. Discussion

This study provides a comprehensive investigation into the nutritional determinants of systemic inflammation in MetS, underscoring critical roles for vitamin D status and dietary intake of fortified foods. We identified robust independent associations of vitamin D deficiency, hypercholesterolemia, and elevated neutrophil-to-lymphocyte ratios with heightened levels of inflammatory biomarkers, specifically C-reactive protein (CRP) and ferritin [54,55]. These biomarkers reflect subclinical inflammation, a hallmark of MetS that contributes substantially to its cardiometabolic complications [7,44]. Previous studies have documented similar associations between hypovitaminosis D and inflammatory activation in MetS; however, few have quantitatively tested mediation models linking dietary intake with systemic inflammation through serum vitamin D levels [56,57,58]. Our findings extend this evidence by demonstrating a statistically significant indirect effect, highlighting a potentially causal pathway that has been hypothesized but rarely quantitatively tested [58,59]. Our data emphasizes the intricate relationship between dietary quality, vitamin D status, and systemic inflammation. Mediation analysis provides empirical evidence that serum vitamin D deficiency mediates the association between insufficient dietary vitamin D intake and increased systemic inflammation, as measured by C-reactive protein and ferritin levels [58,60,61]. The mediation effect suggests that vitamin D deficiency is not merely a marker but a key mechanism promoting chronic inflammation in patients with metabolic syndrome [56,62]. These results support targeted dietary interventions to improve vitamin D intake as a strategy to reduce inflammation and potentially alter the course of metabolic syndrome. Consistently, patients with elevated CRP and ferritin levels are associated with reduced levels of vitamin D-rich foods [63]. Patients with elevated CRP consume less fortified foods such as milk, fish, and traditional cheeses including Oaxaca and Manchego. The use of mediation modeling represents a methodological strength, enabling us to identify vitamin D deficiency not only as an associated factor but as a potential mechanistic link between dietary inadequacy and systemic inflammation [64,65,66,67].

Vitamin D’s potent immunomodulatory functions are well established. It downregulates pro-inflammatory cytokines such as TNF-α and IL-6 and enhances regulatory immune responses, including increased activity of regulatory T cells [68,69]. These actions collectively improve insulin sensitivity, endothelial function, and cardiovascular health. Vitamin D deficiency fosters a pro-inflammatory environment that exacerbates insulin resistance and metabolic derangements, accelerating MetS progression [70,71]. This paradigm is supported by accumulating epidemiological and mechanistic evidence, contextualizing our findings that vitamin D deficiency is a key driver of systemic inflammation measured by CRP and ferritin in our cohort [72,73]. These findings align with the framework of “metainflammation,” the chronic, metabolically driven inflammatory state that characterizes obesity and MetS, in which micronutrient deficiencies—particularly vitamin D—interact with dysregulated immune pathways [74].

Importantly, manchego cheese (derived from sheep’s milk and richer in vitamin D and fat-soluble vitamins A, D, and E) was more strongly protective against vitamin D deficiency than Oaxaca cheese, which is sourced from cow’s milk. This distinction spotlights the necessity to consider the specific vitamin and nutrient profiles of individual foods rather than generalizing food categories when designing dietary recommendations for MetS and inflammatory states [75,76,77,78]. Intriguingly, our analysis uncovered a positive association between butter intake and vitamin D deficiency, suggesting potentially deleterious effects of certain dietary fats on vitamin D metabolism or absorption. This novel observation merits further exploration of how different fat sources and food matrices modulate metabolic and inflammatory pathways within MetS [75,79,80]. A major unmeasured determinant of vitamin D status is sunlight exposure, which can modulate serum 25(OH)D independently of dietary intake. Although sunlight exposure was assessed in this study, it was measured only in a rudimentary manner. Future research should incorporate more precise quantification of solar radiation effects—beyond general exposure metrics—to better disentangle the relative contributions of environmental versus nutritional determinants [81,82].

Adiposity remains a central catalyst of chronic low-grade inflammation. Confirming previous literature, obesity grade 2 or greater was independently linked to elevated CRP, ferritin, and vitamin D deficiency [83,84]. However, we observed a paradoxical inverse association of obesity with hyperferritinemia. This ferritin paradox likely reflects complex iron metabolism within inflamed adipose tissue. Ferritin serves a dual role as an iron storage protein and acute-phase reactant elevated during inflammation [84,85,86]. Importantly, hyperferritinemia and CRP act as complementary, independent inflammatory markers in MetS. Unlike Hb (confounded in multivariable models), ferritin ≥ 200 μg/L independently predicts CRP ≥ 3 mg/L (aOR 8.00, *p* < 0.001; Table 3) after mutual adjustment, while CRP reciprocally predicts ferritin (Table 4: aOR 5.06). This bidirectional relationship reflects reciprocal acute-phase activation rather than unidirectional mediation or iron accumulation, positioning hyperferritinemia as a marker of systemic inflammation rather than iron stores. In MetS, chronic inflammatory cytokines induce ferritin synthesis in multiple cells, including macrophages and adipocytes. Simultaneously, adipose tissue may sequester iron, a process mediated by upregulated hepcidin, leading to altered systemic iron distribution characterized by functional iron deficiency despite high ferritin levels [87,88,89]. This disconnect complicates ferritin’s interpretation as a simple marker of iron status, underscoring its role as an inflammation proxy and potential contributor to metabolic dysfunction through oxidative stress and insulin resistance pathways [90,91]. These nuanced dynamics warrant focused mechanistic studies to elucidate ferritin’s dual roles in MetS pathophysiology. Elevated ferritin may thus contribute to mitochondrial dysfunction, oxidative stress, and impaired insulin signaling, positioning it as an active contributor to metabolic deterioration rather than a passive biomarker [92,93].

Although anemia was infrequent (2.8%), elevated Hb levels (≥14.3 g/dL) strongly predicted hyperferritinemia (aOR 63.23, *p* < 0.001; AUC 0.826) yet did not independently predict CRP in multivariate models. This dissociation—robust Hb-hyperferritinemia association alongside absence of direct Hb-CRP linkage—suggests ferritin may serve as an intermediary linking Hb elevation to systemic inflammation in MetS, consistent with hyperferritinemia’s established role as an acute-phase reactant. The pattern aligns with existing vitamin D → ferritin mediation (Sobel *p* = 0.014, Table 7) and warrants formal testing of a sequential Hb-ferritin-CRP pathway in prospective studies. Prior reports confirm elevated Hb in MetS predicts CV events [94,95,96]. The Hb≥14.3 g/dL cutoff thus emerges as a promising ferritin-linked prognostic marker for inflammatory MetS, meriting validation in longitudinal cohorts. The neutrophil-to-lymphocyte ratio, a readily accessible metric of immune system dysregulation, is associated strongly with CRP and ferritin and is gaining recognition for cardiovascular risk prediction in MetS [97,98]. This underlines the complex immune-inflammatory interplay driving metabolic disease and offers a potential clinical tool for risk stratification and tailored intervention. The heterogeneity observed in inflammatory markers and nutrient intake patterns supports the concept of metabolically distinct phenotypes within MetS. Personalized nutrition approaches may therefore be more effective than uniform dietary recommendations [99,100].

Synthesizing these findings highlights the promise of integrated nutritional strategies emphasizing vitamin D repletion and diets rich in fortified dairy products, fish, and vitamin D-dense foods like Manchego cheese. Such approaches align with evolving precision nutrition paradigms aimed at ameliorating inflammation and metabolic dysfunction. By addressing modifiable dietary factors, these strategies complement pharmacologic lipid-lowering therapies that may confer additional anti-inflammatory benefits, potentially attenuating MetS progression and cardiovascular risk. Given the high population prevalence of MetS and widespread vitamin D insufficiency in Latin America, the implications of our findings extend beyond individual-level dietary counseling. They highlight the need for public health strategies such as food fortification policies, nutrition education, and screening programs for micronutrient deficiencies in metabolic-risk populations.

Limitations of this study include its cross-sectional design limiting causal inference, and reliance on food frequency questionnaires that preclude precise nutrient quantification. Future longitudinal and randomized controlled trials are essential to validate these associations, investigate mechanistic pathways, and test the efficacy of targeted nutritional interventions in reducing inflammation and improving metabolic outcomes. Emerging evidence suggests that gut microbiota composition modulates vitamin D metabolism and inflammatory tone [101,102]. Although not assessed in this study, future work integrating microbiome profiling may clarify whether dysbiosis interacts with vitamin D deficiency to exacerbate systemic inflammation in MetS.

## 5. Conclusions

In conclusion, this study demonstrates that vitamin D deficiency is closely associated with systemic inflammation in patients with metabolic syndrome, as reflected by elevated CRP, ferritin, and neutrophil-to-lymphocyte ratio levels. Mediation analysis provides evidence that serum vitamin D deficiency acts as a key intermediary between insufficient dietary vitamin D intake and increased inflammatory burden. Our findings also highlight the complex role of ferritin as an inflammation-related biomarker rather than a simple indicator of iron stores, underscoring the inflammatory nature of metabolic syndrome. The observed associations between specific fortified foods and vitamin D status suggest that food quality and nutrient composition should be considered when designing nutritional interventions. Collectively, these results support the implementation of targeted nutritional strategies aimed at improving vitamin D levels as a complementary approach to reduce chronic inflammation and cardiometabolic risk in metabolic syndrome. Future longitudinal and interventional studies are warranted to confirm causality and to evaluate the effectiveness of vitamin D–focused dietary interventions in this high-risk population.

## Figures and Tables

**Table 1:** Clinical and Biochemical Characteristics According to High C-Reactive Protein Status in Patients with Metabolic Syndrome.

Variable	All (*n* = 141)	CRP < 3 mg/L (*n* = 77)	CRP ≥ 3 mg/L (*n* = 64)	*p*-Value
Age (years)	49.21 ± 9.85	49.60 ± 9.67	48.73 ± 10.11	0.606
Female (%)	68.10	62.30	75.00	0.146
BMI (kg/m^2^)	33.61 ± 7.92	32.10 ± 7.16	35.42 ± 8.46	0.013
Systolic BP (mmHg)	137.83 ± 18.89	136.27 ± 20.82	139.70 ± 16.24	0.285
Diastolic BP (mmHg)	83.11 ± 11.15	81.75 ± 10.60	84.73 ± 11.65	0.114
Exercise (days/week)	1.42 ± 1.89	1.16 ± 1.80	1.73 ± 1.97	0.071
SEPI Total Score	12.50 ± 4.24	12.70 ± 4.20	12.25 ± 4.31	0.531
Serum Vitamin D (ng/mL)	18.43 ± 7.40	22.09 ± 7.04	14.04 ± 5.08	<0.001
Dietary Vitamin D intake (IU/month)	3068.0 (2259.5–5207.5) ^a^	3676.0 (2490.0–6297.0) ^a^	2604.0 (2010.0–3967.2) ^a^	0.001 *
Hemoglobin (g/dL)	14.11 ± 0.82	13.71 + 0.76	14.59 + 0.63	<0.001
Glucose (mg/dL)	204.29 ± 85.39	214.22 ± 91.46	192.35 ± 76.47	0.131
HDL (mg/dL)	38.59 ± 21.73	38.40 ± 27.51	38.81 ± 11.68	0.913
Total Cholesterol (mg/dL)	237.97 ± 51.74	214.83 ± 49.25	265.81 ± 39.76	<0.001
Triglycerides (mg/dL)	214.0 (156.0–314.5) ^a^	232.0 (155.5–310.5) ^a^	202.5 (167.5–315.2) ^a^	0.791 *
NLR	2.80 ± 1.20	2.47 ± 0.85	3.20 ± 1.42	<0.001

The serum vitamin D levels were compared between the groups with C-reactive protein (CRP) < 3 mg/L and CRP ≥ 3 mg/L, showing means of 22.09 (SD 7.04) versus 14.04 (SD 5.08), respectively. The statistical power for detecting this difference was calculated to be 99% using the sample size and power calculator available at https://clincalc.com/stats/samplesize.aspx (accessed on 30 November 2025) [50]. NLR: Neutrophil-to-Lymphocyte Ratio. This high power confirms the adequacy of the sample size to detect a statistically significant difference in serum vitamin D levels between the CRP groups. * Nonparametric comparisons between groups were performed using the Mann–Whitney U test. ^a^ Data are presented as medians with first (Q1) and third (Q3) quartiles.

**Table 2:** Dietary Intake Variables (Servings per Week) According to High C-Reactive Protein Status in Patients with Metabolic Syndrome.

Food Item	All (*n* = 141)	CRP < 3 mg/L (*n* = 77)	CRP ≥ 3 mg/L (*n* = 64)	*p*-Value
Milk	2.49 ± 1.96	2.90 ± 2.12	2.00 ± 1.63	0.006
Cottage cheese	0.94 ± 0.94	0.97 ± 1.03	0.89 ± 0.84	0.603
Fresh cheese	1.70 ± 1.05	2.00 ± 1.04	1.34 ± 0.95	<0.001
Manchego-type cheese	1.62 ± 1.10	1.82 ± 1.10	1.38 ± 1.06	0.017
Cream	1.12 ± 0.81	1.19 ± 0.86	1.03 ± 0.76	0.237
Yogurt	2.30 ± 2.00	2.69 ± 2.20	1.84 ± 1.63	0.012
Ice cream	1.30 ± 0.70	1.45 ± 0.66	1.13 ± 0.70	0.005
Eggs	3.28 ± 1.65	3.38 ± 1.75	3.17 ± 1.53	0.465
Tuna	1.84 ± 1.23	2.04 ± 1.29	1.59 ± 1.11	0.032
Chicken liver	0.89 ± 0.78	0.99 ± 0.80	0.78 ± 0.74	0.120
Fish	1.86 ± 1.05	2.05 ± 1.16	1.63 ± 0.86	0.016
Seafood	0.96 ± 0.71	1.00 ± 0.74	0.91 ± 0.66	0.434
Butter	1.59 ± 0.99	1.48 ± 0.94	1.72 ± 1.03	0.154
Chicken	2.59 ± 1.44	2.69 ± 1.49	2.47 ± 1.39	0.371
Ham	1.58 ± 1.06	1.36 ± 1.06	1.84 ± 1.01	0.007
Beef	2.80 ± 0.90	2.88 ± 0.86	2.70 ± 0.95	0.241
Pork	2.89 ± 1.01	2.86 ± 0.85	2.92 ± 1.17	0.706
Beans	3.61 ± 2.09	3.38 ± 1.98	3.89 ± 2.20	0.147
Peas	1.00 ± 0.77	0.90 ± 0.66	1.13 ± 0.86	0.077
Green fava beans	0.79 ± 0.69	0.84 ± 0.71	0.72 ± 0.68	0.288
Lentils	1.04 ± 0.83	1.09 ± 0.86	0.98 ± 0.79	0.448
Chickpeas	0.97 ± 0.78	0.94 ± 0.83	1.02 ± 0.72	0.545
Oats	0.99 ± 0.90	0.90 ± 0.91	1.11 ± 0.88	0.161
Spinach/leafy greens	0.92 ± 0.70	0.87 ± 0.68	0.98 ± 0.72	0.335

The serum vitamin D levels were compared between the groups with C-reactive protein (CRP) < 3 mg/L and CRP ≥ 3 mg/L, showing means of 22.09 (SD 7.04) versus 14.04 (SD 5.08), respectively. The statistical power for detecting this difference was calculated to be 99% using the sample size and power calculator available at https://clincalc.com/stats/samplesize.aspx (accessed on 30 November 2025). This high power confirms the adequacy of the sample size to detect a statistically significant difference in serum vitamin D levels between the CRP groups.

**Table 3:** Factors Associated with High C-Reactive Protein (CRP ≥ 3 mg/L) in Metabolic Syndrome: Bivariate and Multivariable Analysis.

Variable	Bivariate OR	95% CI	*p*-Value	Adjusted OR	95% CI	*p*-Value
Age ≥ 54 years	1.06	0.54–2.06	0.866	—	—	—
Female sex	1.81	0.87–3.76	0.110	—	—	—
≥3 days/week of exercise	1.40	0.69–2.85	0.351	—	—	—
SEPI score ≥ median (≥12)	0.91	0.47–1.76	0.770	—	—	—
Obesity grade II or higher	2.44	1.16–5.15	0.019	—	—	—
Vitamin D deficiency (<20 ng/mL)	15.97	6.39–39.92	<0.001	7.11	2.59–19.48	<0.001
Vitamin D intake < 3281 IU/month	0.56	0.28–1.10	0.094	—	—	—
Hemoglobin ≥ 14.3 g/L	10.65	4.84–23.46	<0.001	—	—	—
Ferritin ≥ 200 mg/L	16.25	7.04–37.50	<0.001	8.00	3.21–19.91	<0.001
High systolic BP	1.31	0.65–2.62	0.450	—	—	—
High diastolic BP	1.53	0.78–3.03	0.217	—	—	—
Hypertriglyceridemia	1.05	0.45–2.44	0.913	—	—	—
Hyperglycemia	1.05	0.45–2.44	0.913	—	—	—
Total cholesterol ≥ 200 mg/dL	8.06	3.11–20.89	<0.001	—	—	—
Low HDL	0.72	0.27–1.88	0.497	—	—	—
NLR ≥ 3.5	2.76	1.26–6.05	0.011	—	—	—

Bivariate and multivariable logistic regression analyses identifying factors associated with high CRP in patients with metabolic syndrome. Variables with *p* < 0.05 in the bivariate model were entered into the multivariable model using backward stepwise selection. Adjusted odds ratios (AdOR) represent independent associations after controlling demographic and clinical covariates. SEPI: Sun Exposure and Protection Index (SEPI), where a higher score generally indicates greater exposure to sunlight.

**Table 4:** Factors Associated with High Ferritin Levels (≥200 ng/mL) in Metabolic Syndrome: Bivariate and Multivariable Analysis.

Variable	Bivariate OR	95% CI	*p*-Value	Adjusted OR	95% CI	*p*-Value
Age ≥ 54 years	0.73	0.37–1.44	0.364	—	—	—
Female sex	1.02	0.50–2.09	0.957	—	—	—
≥3 days/week of exercise	1.12	0.55–2.29	0.756	—	—	—
SEPI score ≥ median (≥12)	0.63	0.32–1.24	0.181	—	—	—
Obesity grade II or higher	1.08	0.52–2.25	0.836	0.11	0.02–0.60	0.011
Vitamin D deficiency (<20 ng/mL)	16.14	6.19–42.06	<0.001	24.69	3.76–162.16	0.001
Vitamin D intake < 3281 IU/month	0.55	0.28–1.08	0.084	—	—	—
Hemoglobin ≥ 14.3 g/L	51.70	18.69–143.01	<0.001	63.23	13.00–307.50	<0.001
CRP ≥ 3 mg/L	16.25	7.04–37.50	<0.001	5.06	1.38–18.47	0.014
High systolic BP	1.24	0.62–2.50	0.547	—	—	—
High diastolic BP	1.15	0.58–2.28	0.684	—	—	—
Hypertriglyceridemia	0.91	0.39–2.12	0.825	—	—	—
Hyperglycemia	0.88	0.26–3.03	0.839	—	—	—
Total cholesterol ≥ 200 mg/dL	16.79	4.86–58.04	<0.001			
Low HDL	0.38	0.14–1.03	0.057			
NLR ≥ 3.5	2.37	1.09–5.13	0.028			

Bivariate and multivariable logistic regression analyses identifying factors associated with high ferritin levels (≥200 ng/mL) in patients with metabolic syndrome. Variables with *p* < 0.05 in the bivariate model were entered into the multivariable model using backward stepwise selection. Adjusted odds ratios (AdOR) represent independent associations after controlling for demographic and clinical covariates. SEPI: Sun Exposure and Protection Index (SEPI), where a higher score generally indicates greater exposure to sunlight.

**Table 5:** Factors Associated with Vitamin D Deficiency in Metabolic Syndrome: Bivariate and Multivariable Analysis.

Variable	Bivariate Model OR	95% CI	*p*-Value	Multivariable Model AdOR	95% CI	*p*-Value
Female	1.40	0.68–2.85	0.362	—	—	—
Age ≥ 54 years	0.60	0.31–1.19	0.145	—	—	—
Obesity grade II or higher	4.12	1.73–9.81	0.001	—	—	—
≥3 days/week of exercise	1.23	0.59–2.54	0.579	—	—	—
SEPI ≥ median (≥12)	0.49	0.25–0.97	0.040	0.38	0.16–0.92	0.033
Milk ≥ 3 servings/week	0.16	0.08–0.34	<0.001	0.37	0.14–0.96	0.040
Oaxaca cheese ≥ 3 servings/week	0.17	0.07–0.39	<0.001	—	—	—
Manchego/cottage-type cheese ≥ 3 servings/week	0.09	0.03–0.26	<0.001	0.20	0.06–0.68	0.010
Cream cheese ≥ 3 servings/week	0.18	0.04–0.90	0.037	—	—	—
Yogurt ≥ 3 servings/week	0.18	0.09–0.38	<0.001	—	—	—
Ice cream ≥ 3 servings/week	0.34	0.03–3.86	0.385	—	—	—
Eggs ≥ 3 servings/week	0.72	0.36–1.45	0.358	—	—	—
Seafood ≥ 3 servings/week	1.00	1.00–1.00	0.999	—	—	—
Butter ≥ 3 servings/week	3.37	1.18–9.58	0.023	4.36	1.18–16.16	0.027
Chicken ≥ 3 servings/week	0.75	0.38–1.47	0.404	—	—	—
Ham ≥ 3 servings/week	1.33	0.56–3.14	0.516	—	—	—
Beef ≥ 3 servings/week	0.39	0.17–0.88	0.023	—	—	—
Pork ≥ 3 servings/week	0.49	0.22–1.08	0.077	—	—	—
Beans ≥ 3 servings/week	0.91	0.45–1.85	0.788	—	—	—
Fava beans ≥ 3 servings/week	1.00	1.00–1.00	0.999	—	—	—
Lentils ≥ 3 servings/week	0.68	0.16–2.85	0.602	—	—	—
Chickpeas ≥ 3 servings/week	1.05	0.17–6.49	0.958	—	—	—
Oats ≥ 3 servings/week	3.65	0.42–32.13	0.243	—	—	—
Spinach ≥ 3 servings/week	1.00	1.00–1.00	0.999	—	—	—
Cottage cheese ≥ 3 servings/week	0.08	0.01–0.63	0.017	—	—	—
Tuna ≥ 3 servings/week	0.17	0.07–0.41	<0.001	—	—	—
Fish ≥ 3 servings/week	0.07	0.03–0.19	<0.001	0.15	0.05–0.45	0.001
Green peas ≥ 3 servings/week	1.00	1.00–1.00	0.999	—	—	—

Bivariate and multivariable logistic regression analyses evaluating factors associated with vitamin D deficiency (<20 ng/mL) in patients with metabolic syndrome. Variables with *p* < 0.05 in bivariate analysis were entered into the multivariable model using backward stepwise selection. Adjusted odds ratios (AdOR) represent independent associations after adjustment for demographic, clinical, and dietary covariates. SEPI: Sun Exposure and Protection Index (SEPI), where a higher score generally indicates greater exposure to sunlight.

**Table 6:** Serum Vitamin D Deficiency Mediates the Association Between Dietary Vitamin D Intake and Elevated C-Reactive Protein in Metabolic Syndrome.

Model	Independent Variable	Dependent Variable	B Coefficient	Standard Error	OR	95% CI for OR	*p*-Value	Comments
Model A *	Deficient Vitamin D Intake	Vitamin D Deficiency	2.036	0.538	7.662	2.669–21.995	<0.001	“a” Path
Model B **	Vitamin D Deficiency	High CRP	2.462	0.768	11.731	2.603–52.875	0.001	“b” Path, adjusted for X
Direct Effect ***	Deficient Vitamin D Intake	High CRP	−0.616	0.484	0.54	0.209–1.394	0.203	Direct effect with mediator included
Indirect Effect (Sobel Test)	–	–	2.446	2.049	–	–	0.014	Mediation effect

Mediation analysis evaluating serum vitamin D deficiency as a mediator between deficient dietary vitamin D intake and elevated C-reactive protein (CRP) levels in individuals with metabolic syndrome. Logistic regression models were used to estimate indirect (“a × b”) and direct effects, and statistical significance of the mediation was confirmed with the Sobel test. * Model A adjusted for: female sex, age ≥ 54 years, obesity grade II or higher, ≥3 days/week of exercise, SEPI ≥ 12, hemoglobin > 14.3 g/L, ferritin ≥ 200 mg/L, CRP ≥ 3 mg/L, and NLR ≥ 3.5. ** Model B adjusted for: deficient monthly vitamin D intake, female sex, age ≥ 54 years, obesity grade II or higher, ≥3 days/week of exercise, hemoglobin > 14.3 g/L, ferritin ≥ 200 mg/L, and NLR ≥ 3.5. *** Direct effect adjusted for serum vitamin D deficiency. High Ferritin: ≥ 200 μg/L; High Hb: Hemoglobin ≥ 14.3 g/dL; High CRP: C-Reactive Protein ≥ 3 mg/L.

**Table 7:** Serum Vitamin D Deficiency Mediates the Association Between Dietary Vitamin D Intake and High Ferritin in Metabolic Syndrome.

Model	Independent Variable	Dependent Variable	B Coefficient	Standard Error	OR	95% CI for OR	*p*-Value	Comments
Model A *	Deficient Vitamin D Intake	Vitamin D Deficiency	2.036	0.538	7.662	2.669–21.995	<0.001	“a” Path
Model B **	Vitamin D Deficiency	High Ferritin	3.013	0.947	20.357	3.181–130.255	0.001	“b” Path, adjusted for X
Direct Effect ***	Deficient Vitamin D Intake	High Ferritin	−0.517	0.470	0.597	0.238–1.498	0.272	Direct effect with mediator included
Indirect Effect (Sobel Test)	–	–	2.435	2.518	–	–	0.014	Mediation effect

Mediation analysis evaluating serum vitamin D deficiency as a mediator between deficient dietary vitamin D intake and High Ferritin levels in individuals with metabolic syndrome. Logistic regression models were used to estimate indirect (“a × b”) and direct effects, and statistical significance of the mediation was confirmed with the Sobel test. * Model A adjusted for: female sex, age ≥ 54 years, obesity grade II or higher, ≥3 days/week of exercise, SEPI ≥ 12, hemoglobin > 14.3 g/L, ferritin ≥ 200 mg/L, CRP ≥ 3 mg/L, and NLR ≥ 3.5. ** Model B adjusted for: deficient monthly vitamin D intake, female sex, age ≥ 54 years, obesity grade II or higher, ≥3 days/week of exercise, hemoglobin > 14.3 g/L, CRP ≥ 3 mg/L, and NLR ≥ 3.5. *** Direct effect adjusted for serum vitamin D deficiency. High Ferritin: ≥ 200 μg/L; High Hb: Hemoglobin ≥ 14.3 g/dL; High CRP: C-Reactive Protein ≥ 3 mg/L.

**Table 8:** Ferritin Mediates the Association Between Hemoglobin and C-Reactive Protein in Metabolic Syndrome.

Model	Independent Variable	Dependent Variable	B Coefficient	Standard Error	OR	95% CI for OR	*p*-Value	Comments
Model A *	High Hb	High Ferritin	4.147	0.807	63.238	13.005–307.502	<0.001	“a” Path
Model B **	High Ferritin	High CRP	2.079	0.465	8.000	3.213–19.918	<0.001	“b” Path, adjusted for X
Direct Effect ***	High Hb	High CRP	0.993	0.562	2.541	0.845–7.645	0.097	Direct effect with mediator included
Indirect Effect (Sobel Test)	–	–	3.373	2.556	–	–	<0.001	Mediation effect

Mediation analysis evaluating serum ferritin as a mediator between elevated hemoglobin and elevated C-reactive protein (CRP) levels in individuals with metabolic syndrome. Logistic regression models were used to estimate indirect (“a × b”) and direct effects, and statistical significance of the mediation was confirmed with the Sobel test. * Model A adjusted for: female sex, age ≥ 54 years, obesity grade II or higher, ≥3 days/week of exercise, SEPI ≥ 12, deficient vitamin D intake, serum vitamin D deficiency, CRP ≥ 3 mg/L, and NLR ≥ 3.5. ** Model B adjusted for: deficient monthly vitamin D intake, female sex, age ≥ 54 years, obesity grade II or higher, ≥3 days/week of exercise, deficient vitamin D intake, serum vitamin D deficiency, SEPI ≥ 12, High Hb, and NLR ≥ 3.5. *** Direct effect adjusted for serum high Ferritin. High Ferritin: ≥200 μg/L; High Hb: Hemoglobin ≥ 14.3 g/dL; High CRP: C-Reactive Protein ≥ 3 mg/L.

## Data Availability

The original contributions presented in the study are included in the article; further inquiries can be directed to the corresponding authors.
